# The Activity of Liposomal Linolenic Acid Against *Helicobacter pylori In Vitro* and Its Impact on Human Fecal Bacteria

**DOI:** 10.3389/fcimb.2022.865320

**Published:** 2022-05-17

**Authors:** Ya Wang, Shuang Wu, Le Wang, Youhua Wang, Dongsheng Liu, Yingjun Fu, Yong Xie

**Affiliations:** ^1^ Key Laboratory of Digestive Diseases of Jiangxi, Department of Gastroenterology, The First Affiliated Hospital of Nanchang University, Nanchang, China; ^2^ School of Pharmacy, Nanchang University, Nanchang, China

**Keywords:** *Helicobacter pylori*, liposomal linolenic acid, antibacterial action, human fecal bacteria, *in vitro*

## Abstract

*Helicobacter pylori* (*H. pylori*) infection is associated with a variety of gastrointestinal diseases. Here, we focused on the activity of a novel nanomedicine-liposomal linolenic acid (LipoLLA) against *H. pylori* and its impact on human fecal bacteria *in vitro*. The minimum inhibitory concentrations (MICs) of LipoLLA against 30 *H. pylori* clinical strains were determined in combination with amoxicillin (AMX), metronidazole (MTZ), levofloxacin (LVFX) and clarithromycin (CAM). Bactericidal activity was measured by generating concentration-bactericidal curves at different times and pH values. Leakage of glucose (GLU) and aspartate aminotransferase (AST) was detected, combined with detection of changes in morphology by electron microscopy, to study the mechanism of action of LipoLLA against *H. pylori*. The effect of LipoLLA on human fecal bacteria was studied by high-throughput sequencing of fecal samples. We observed a synergistic or additive effect when LipoLLA was combined with AMX, MTZ, LVFX and CAM. The concentration-sterilization curves were pH and time dependent. After treatment with LipoLLA, GLU and AST levels were increased (P<0.05), and the morphology of *H. pylori* changed significantly. Moreover, LipoLLA activity led to no significant changes in the intestinal flora in terms of alpha diversity, species composition, beta diversity, etc. In conclusion, LipoLLA showed good anti-*H. pylori* effects. It destroyed the outer membrane barrier and caused leakage of the bacterial contents to achieve anti-*H. pylori* effects. And LipoLLA had little effect on human fecal bacteria *in vitro*.

## Introduction

*Helicobacter pylori* (*H. pylori*) infection can cause a series of gastrointestinal diseases, including chronic gastritis, peptic ulcers, and gastric cancer (Schlaermann et al., 2016). In addition, studies have proven that some extragastric diseases, such as iron-deficiency-related anemia and vitamin B12 deficiency, are also associated with *H. pylori* infection (Ražuka-Ebela et al., 2018 ). Successful eradication of *H. pylori* can reduce the risk of related diseases and benefit patients to varying degrees. A study showed that among *H. pylori*-positive duodenal ulcer patients, the recurrence rate of gastric ulcers and duodenal ulcers in patients receiving *H. pylori* eradication therapy was significantly lower than that in patients who did not receive eradication therapy (Ford et al., 2016). In addition, eradication of *H. pylori* can also prevent indigestion and other symptoms (Moayyedi et al., 2017). Currently, the recommended *H. pylori* eradication therapy is the quadruple therapy consisting of a bismuth agent and a proton pump inhibitor (PPI) combined with two antibacterial drugs in China (Malfertheiner et al., 2017; Liu et al., 2018). The two antibacterial drugs used in this combination include amoxicillin (AMX), metronidazole (MTZ), levofloxacin (LVFX), clarithromycin (CAM), tetracycline and furazolidone. With the increasing resistance of *H. pylori* to antibacterial drugs, especially MTZ, LVFX and CAM (Zhang, 2015; Savoldi et al., 2018), the optimal overall eradication rate of *H. pylori* is difficult to reach (Graham et al., 2007). In addition, frequent adverse reactions and poor patient compliance also result in therapy failure. The use of PPIs and antibiotics may also disturb the intestinal flora (Hsu et al., 2018). To solve these problems, the study of effective non-antibiotics has become a research hotspot.

Linolenic acid (LLA) is a polyunsaturated fatty acid. Studies have shown that LLA could inhibit the proliferation of *H. pylori in vitro* but not *in vivo*, possibly because of the low solubility of LLA and its tendency to oxidize, esterify, and form complexes with lipoproteins *in vivo* (Jung and Lee, 2016). To overcome this challenge, LLA was packed into liposome, and the liposomal LLA (LipoLLA) prepared thereby showed good anti-*H. pylori* activity (Obonyo et al., 2012; Thamphiwatana et al., 2014).

In this study, we further elucidated the combined effects of LipoLLA with AMX, MTZ, LVFX and CAM. Meanwhile, the effects of pH and time on the bactericidal effect of LipoLLA were studied. As well, we evaluated the effect of LipoLLA on the outer membrane barrier of *H. pylori*. We have also discussed the effects of LipoLLA on probiotics and the human intestinal flora to provide a theoretical basis for the efficacy and safety of LipoLLA as an *H. pylori* eradication therapy in the future.

## Materials and Methods

### Preparation of LipoLLA

LipoLLA was prepared by a standard vesicle extrusion method as previously described (Thamphiwatana et al., 2014). Briefly, phospholipids, cholesterol and LLA were mixed in a weight ratio of 6:1:3 (total weight of 16 mg), and dissolved in chloroform (4 mL). Then chloroform was evaporated to form a thin layer of lipid. After hydration, vortexing and sonication, the resulting lipid vesicles were extruded by microextruder through a polycarbonate membrane with a pore size of 100 nm. Following, remove unloaded LLA and sterilize through a 0.22 μm filter. Finally, the hydrodynamic size and surface zeta-potential of liposomes were measured to ensure the stability. The prepared LipoLLA were stored at 4°C.

### Bacterial Strains

The *H. pylori* strains ATCC43504 (NCTC11637) and SS1 and thirty clinical isolates were obtained from the Department of Gastroenterology, The First Affiliated Hospital of Nanchang University. The research protocol was approved by the Ethics Committee of the First Affiliated Hospital of Nanchang University (IRB 2018-116). *Lactobacillus* BG-2-2 and *Bifidobacterium* WBIN03 were kindly provided by Professor Wei Hua, Sino German Joint Research Institute, Nanchang University. *H. pylori* strains were maintained on Campylobacter agar base (OXOID, UK) plates supplemented with 5% sheep blood under microaerobic conditions (5% O2, 10% CO2, and 85% N2) at 37°C for 2–3 days. The *Lactobacillus* strain was grown in MRS medium (Solarbio Technology Co., LTD, Beijing, China) under anaerobic conditions. The *Bifidobacterium* strain was grown in MRS medium containing 0.05% L-cysteine hydrochloride monohydrate under anaerobic conditions.

### The Minimum Inhibitory Concentrations (MICs) of LipoLLA and Antimicrobial Agents Against *H. pylori*

The MICs of LipoLLA and four antibiotics (AMX, MTZ, LVFX and CAM) against *H. pylori* were detected by the agar dilution method according to the Clinical and Laboratory Standards Institute (Cockerill, 2012). Briefly, serial 2-fold dilutions of the drugs were prepared in Mueller-Hinton agar. The bacteria were harvested, resuspended in 1 mL of physiological saline, and adjusted to an optical density at 600 nm of 1×10^7^-1×10^8^ colony forming unit (CFU)/mL. Then 10 μL of the test bacterial suspension was inoculated on the plate at 37°C under microaerobic conditions for 72 h. ATCC43504 was used as quality control strain. The MIC value was the lowest concentration that led to complete inhibition of bacterial growth compared with the blank control group. The resistance of the strains to the four antibiotics was determined: AMX≥1 μg/mL, MTZ≥ 8 μg/mL, LVFX≥1 μg/mL, and CAM≥1 μg/mL.

### Combined Effects of LipoLLA and Four Antimicrobial Agents

According to the MIC value of each drug when used alone, the chessboard method was used to design the concentration combinations of LipoLLA with AMX, MTZ, LVFX and CAM and to determine the MIC values for combined use (Odds, 2003). Serial 2-fold dilutions of LipoLLA with the four antibiotics were mixed in a 1:1 ratio in Mueller-Hinton agar. Then the bacteria were similarly prepared as test suspension and incubated on the agar at 37°C under microaerobic conditions for 72 h before determining MICs. The combinatorial effect was determined by the fractional inhibitory concentration index (FICI) (Isenberg, 1992).


$$\text{FICI=}\frac{{\text{MIC}}_{A1}}{{\text{MIC}}_{A2}}+\frac{{\text{MIC}}_{B1}}{{\text{MIC}}_{B2}}$$

Where MIC_A1_ is the MIC of LipoLLA combined with antibiotics; MIC_A2_ is the MIC of LipoLLA alone; MIC_B1_ is the MIC of antibiotics combined with LipoLLA; and MIC_B2_ is the MIC of antibiotics alone. When FICI ≤ 0.5, the combinatorial effect of two drugs was defined as synergistic; when 0.5<FICI ≤ 1, the effect was defined as additive; when 1<FICI ≤ 2, the effect was defined as irrelevant; and when FICI>2, the combinatorial effect was defined as antagonistic (Shang et al., 2019).

### Concentration Sterilization Curve of LipoLLA Against *H. pylori*

Eight strains of *H. pylori* with different drug resistance properties (SS1, sensitive strain, MTZ-resistant strain, LVFX-resistant strain, CAM-resistant strain, dual drug-resistant strain, triple drug-resistant strain, and quadruple drug-resistant strain) were used in this study. *H. pylori* cells were harvested, and the concentration was adjusted to an optical density at 600 nm of 5×10^6^ CFU/mL, then inoculated into Brucella broth containing 5% (vol/vol) fetal bovine serum (FBS) mixed with different concentrations of LipoLLA at 37°C under microaerobic conditions for 18 h. A series of 10-fold dilutions of the bacterial suspension was prepared, and 100 µL from each diluted sample was inoculated onto a Campylobacter agar base plate supplemented with 5% sheep blood. The plates were cultured in an incubator for 72 h before the number of viable colonies was counted.

### Influence of pH and Time on the Bactericidal Effect of LipoLLA

To research the influence of pH on the anti- *H. pylori* effect of LipoLLA, Brucella broth was adjusted to different solutions at pH 5, 6 and 7 respectively by using concentrated hydrochloric acid. SS1 and a multidrug-resistant strain (MDR2) were used. The bacteria cells were harvested and adjusted to an optical density at 600 nm of 5×10^6^ CFU/mL, then inoculated into Brucella broth containing 5% (vol/vol) FBS mixed with different concentrations of LipoLLA at 37°C under microaerobic conditions for 18 h. After that, a series of 10-fold dilutions of the bacterial suspension was prepared, and 100 µL from each diluted sample was inoculated onto a Campylobacter agar base plate supplemented with 5% sheep blood. The plates were cultured in an incubator for 72 h before the number of viable colonies was counted. Time-kill assay was performed to explore the impact of time on the bactericidal effect of LipoLLA. SS1 was co-cultured with LipoLLA in the same way as above. Then liquid cultures of LipoLLA with different concentrations and SS1 were diluted and inoculated on solid medium at different time points (0 h, 1 h, 2 h, 4 h, 8 h, 12 h and 24 h) for 72 h. The number of viable colonies was counted to obtain the time sterilization curves with different concentrations of LipoLLA.

### Effect of LipoLLA on the Outer Membrane Barrier and Ultrastructure of *H. pylori*

SS1 was inoculated into Brucella broth containing 5% (vol/vol) FBS and cultured with LipoLLA (7.5 μg/mL) for 24 h. Then, the bacteria were harvested by centrifugation at 3000 rpm for 10 min, and the contents of GLU and AST in the supernatant were detected by an automatic biochemical analyzer (HITACHI, Japan) to study the effect of LipoLLA on the outer membrane barrier of *H. pylori*. The remaining pellets treated with LipoLLA (7.5 μg/mL) were prepared for observing the ultrastructure of the strain by scanning electron microscopy (SEM) and transmission electron microscopy (TEM). Briefly, pellets were fixed with 2% glutaraldehyde for 2 h at room temperature. The SEM samples were post fixed with 1% osmium acid in phosphate buffer (0.1 M, pH 7.4), dehydrated, dried by critical point drier (Quorum, K850, UK), and then sprayed with gold over 30 s by carbon coater (IXRF, MSP-2S, USA) before SEM (HITACHI, SU8100, Japan) imaging. To prepare the TEM samples, the fixed bacteria were resuspended in 1% agarose after centrifugation, then post fixed with 1% osmium acid in phosphate buffer (0.1 M, pH 7.4) for 2 h; After dehydration, infiltration and embedding, ultramicrotome (Leica, Leica UC7, Germany) was used for slicing to 60-80 nm sections, and then dried overnight after double-staining with uranium lead. Finally, sections were examined by TEM (HITACHI, HT7700, Japan).

### Bactericidal Effect of LipoLLA on Probiotics

*Lactobacillus* strain BG-2-2 and *Bifidobacterium* WBIN03 were used in this part of study. Bacteria were harvested, and the concentration was adjusted to an optical density at 600 nm of 5×10^6^ CFU/mL. Then, the strains were inoculated into MRS broth (the *Bifidobacterium* strain was grown in MRS medium containing 0.05% L-cysteine hydrochloride monohydrate) mixed with different concentrations of LipoLLA, grown at 37°C under anaerobic conditions (an anaerobic jar and immediately put in an anaerobic bag) for 18 h. Then a series of 10-fold dilutions of the bacterial suspension was prepared, and 100 µL from each diluted sample was inoculated onto an MRS solid medium (0.05% L-cysteine hydrochloride monohydrate was supplemented for the strain WBIN03). The plates were cultured in an incubator for 72 h before the number of viable colonies was counted.

### Effect of LipoLLA on Human Fecal Flora

The research protocol was approved by the Ethics Committee of the First Affiliated Hospital of Nanchang University (IRB 2014-032). And the participant provided written informed consent to participate in this study. The feces of a male, 25 years old, healthy volunteer were collected, stirred with normal saline (add 200 mL saline to every 50 g of feces), then filtered, and centrifugated at 3000 rpm for 5 minutes. Repeating the above filtration and centrifugation, and the obtained mixture were divided into four groups to receive different treatment (n=3, V=20 mL) for the following treatment: the blank control group, the LipoLLA (7.5 μg/mL) group, the AMX (1 μg/mL) group, and the CAM (1 μg/mL) group. All four groups were incubated at 37°C under anaerobic conditions for 24 h. Then centrifugated at 3000 rpm for 5 minutes to obtain the precipitate and placed in a liquid nitrogen environment for 20 min. Finally, samples were all immediately stored in sterile containers and frozen at -80°C until RNA extraction. RNA was extracted using the E.Z.N.A.^®^ Soil RNA Midi Kit and transcribed to cDNA with HiScript^®^ II Q RT SuperMix for qPCR (+gDNA wiper) for polymerase chain reaction (PCR) amplification. The V3-V4 hypervariable regions of the bacterial 16S rRNA gene were amplified with the primers 338F (5’-ACTCCTACGGGAGGCAGCAG-3’) and 806R (5’-GGACTACHVGGGTWTCTAAT-3’) by a thermocycling PCR system (GeneAmp 9700, ABI, USA). The resulting PCR products were purified using the AxyPrep DNA Gel Extraction Kit (Axygen Biosciences, Union City, CA, USA) and quantified using QuantiFluor™-ST (Promega, USA). Purified amplicons were pooled in equimolar amounts and subjected to paired-end sequencing on an Illumina MiSeq platform (Illumina, San Diego, USA) according to the standard protocols recommended by Majorbio Bio-Pharm Technology Co., Ltd. (Shanghai, China).

### Statistical Analysis

Logarithms of the MIC values were taken when LipoLLA, AMX, MTZ, LVFX and CAM were used alone or in combination against *H. pylori*. Student’s t test was used when the difference between groups was normally distributed; otherwise, the Wilcoxon symbol rank sum test was used. One-way analysis of variance was used to compare more than two groups. P ≤ 0.05 was considered statistically significant.

## Results

### Antibacterial Effect of LipoLLA on *H. pylori*

**Figure 1:**
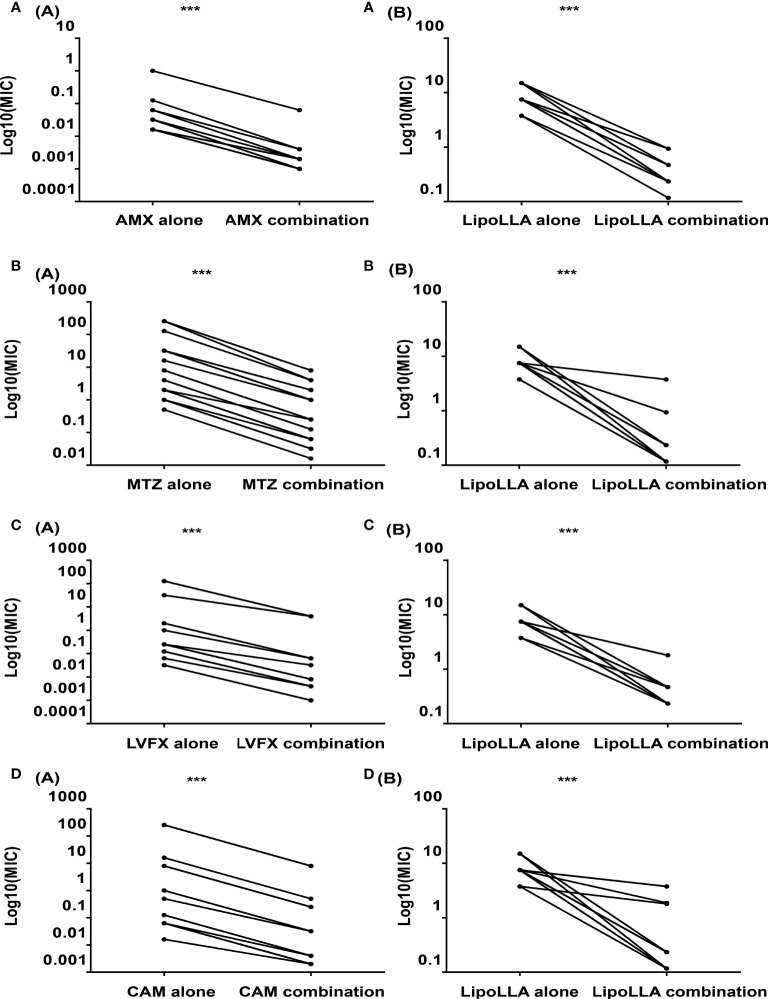
Synergistic effects of LipoLLA with antibiotics against H. pylori. The MICs of LipoLLA and four antibiotics against H. pylori were significantly decreased after the combination of LipoLLA with AMX, MTZ, LVFX and CAM respectively. **(A)** After AMX was used in combination with LipoLLA, MICs of AMX(A(A)) and LipoLLA(A(B)) were significantly reduced compared with the two alone. **(B)** When LipoLLA was combined with MTZ, the MICs of MTZ(B(A)) and LipoLLA(B(B)) decreased significantly with alone. **(C)** When LipoLLA was combined with LVFX, MICs of LVFX(C(A)) and LipoLLA(C(B)) were significantly reduced compared with the two alone. **(D)** After CAM combined with LipoLLA, the MICs of CAM(D(A)) and LipoLLA(D(B)) decreased significantly with alone. AMX (alone), the MICs of amoxicillin against 30 H. pylori strains when used alone; AMX (combination), the MICs of amoxicillin against 30 H. pylori strains when combined with LipoLLA; LipoLLA (alone), the MICs of LipoLLA against 30 H. pylori strains when used alone; LipoLLA (combination), the MICs of LipoLLA against 30 H. pylori strains when combined with amoxicillin. ***P<0.001.

As a quality control strain, MICs of ATCC43504 to four antibacterial drugs AMX, MTZ, LVFX and CAM were all within the quality control range according to CLSI (**Supplementary Table S1**), which proved the experimental results to be reliable. The MICs of LipoLLA and four antibiotics against 30 *H. pylori* strains are shown in **Table 1**. Five strains were sensitive to all four antibiotics (S1-S5), 6 strains were resistant to MTZ (MTZ1-MTZ6), 5 strains were resistant to LVFX (LVFX1-LVFX5), 5 strains were resistant to CAM (CAM1-CAM5), 3 strains exhibited dual drug resistance (DR1-DR3), and 6 strains were multidrug resistant (MDR1-MDR6). For all these strains, the range of the MIC of LipoLLA was 3.75-15 μg/mL, and there was no difference between susceptible and resistant strains (P>0.05). The combined effects of LipoLLA and the four antibiotics were tested on these 30 *H. pylori* clinical isolates. According to the FICIs, LipoLLA significantly lowered the MICs of AMX, MTZ, LVFX and CAM against *H. pylori*. In these 30 *H. pylori* strains, the synergistic effect of LipoLLA combined with AMX was 100% (**Supplementary Table S2**), and the MICs of the two antibacterial agents against *H. pylori* were significantly decreased (P<0.001) (**Figure 1A**). When LipoLLA was combined with MTZ, one strain (3.3%) showed an additive effect, and the others (96.7%) showed a synergistic effect (**Supplementary Table S3**); the MICs decreased significantly (P<0.001) (**Figure 1B**). All 30 tested strains showed synergistic effects when LipoLLA was combined with LVFX (**Supplementary Table S4**), and there were significant differences in MIC reduction after combined use (P<0.001) (**Figure 1C**). After CAM was used in combination with LipoLLA, four strains (13.3%) showed additive effects, and the others (86.7%) showed synergistic effects (**Supplementary Table S5**). The differences in MIC values before and after combined use were still significant (P<0.001) (**Figure 1D**). All of these results indicated that LipoLLA had synergistic anti-*H. pylori* effects when combined with AMX, MTZ, LVFX and CAM *in vitro*.

**Table 1:** MICs of LipoLLA, AMX, MTZ, LVFX and CAM against *H. pylori* strains.

strains	MIC(μg/mL)				
	LipoLLA	AMX	MTZ	LVFX	CAM
S1	7.500	0.016	1.000	0.250	0.063
S2	7.500	0.016	1.000	0.125	0.063
S3	7.500	0.016	4.000	0.032	0.125
S4	7.500	0.016	1.000	0.032	0.063
S5	7.500	0.032	2.000	0.250	0.063
MTZ1	7.500	0.016	8.000	0.032	0.063
MTZ2	7.500	0.063	16.000	0.125	0.063
MTZ3	7.500	0.032	32.000	0.250	0.500
MTZ4	7.500	0.016	32.000	0.250	0.063
MTZ5	7.500	0.016	128.000	0.250	0.063
MTZ6	7.500	0.016	256.000	0.250	0.063
LVFX1	15.000	0.032	0.500	32.000	0.125
LVFX2	7.500	0.016	4.000	128.000	0.063
LVFX3	15.000	0.016	2.000	128.000	0.063
LVFX4	15.000	0.016	4.000	128.000	0.016
LVFX5	7.500	0.016	1.000	128.000	0.063
CAM1	7.500	0.032	2.000	0.125	8.000
CAM2	7.500	0.032	2.000	0.250	8.000
CAM3	7.500	0.016	2.000	0.125	256.000
CAM4	7.500	0.016	2.000	0.063	256.000
CAM5	7.500	0.016	2.000	0.125	256.000
DR1	7.500	0.016	2.000	2.000	1.000
DR2	3.750	0.016	8.000	0.250	1.000
DR3	7.500	0.016	32.000	1.000	0.063
MDR1	3.750	0.016	8.000	128.000	8.000
MDR2	7.500	0.016	32.000	128.000	8.000
MDR3	7.500	0.016	32.000	32.000	8.000
MDR4	7.500	0.063	32.000	2.000	16.000
MDR5	7.500	0.125	256.000	128.000	8.000
MDR6	7.500	1.000	256.000	128.000	256.000

The MICs of 30 clinically isolated H. pylori strains were determined. 5 sensitive strains (S1-S5), 6 MTZ-resistant strains (MTZ1-MTZ6), 5 LVFX- resistant strains (LVFX1-LVFX5), 5 were CAM-resistant strains (CAM1-CAM5), 3 dual drug-resistance strains (DR1-DR3), and 6 multidrug resistant strains (MDR1-MDR6). MIC, minimum inhibitory concentration; AMX, amoxicillin; MTZ, metronidazole; LVFX, levofloxacin; CAM, clarithromycin; S, sensitive strain; DR, dual drug-resistant strain; MDR, multidrug-resistant strain.

### Concentration Sterilization Curve of LipoLLA Against *H. pylori*

**Figure 2:**
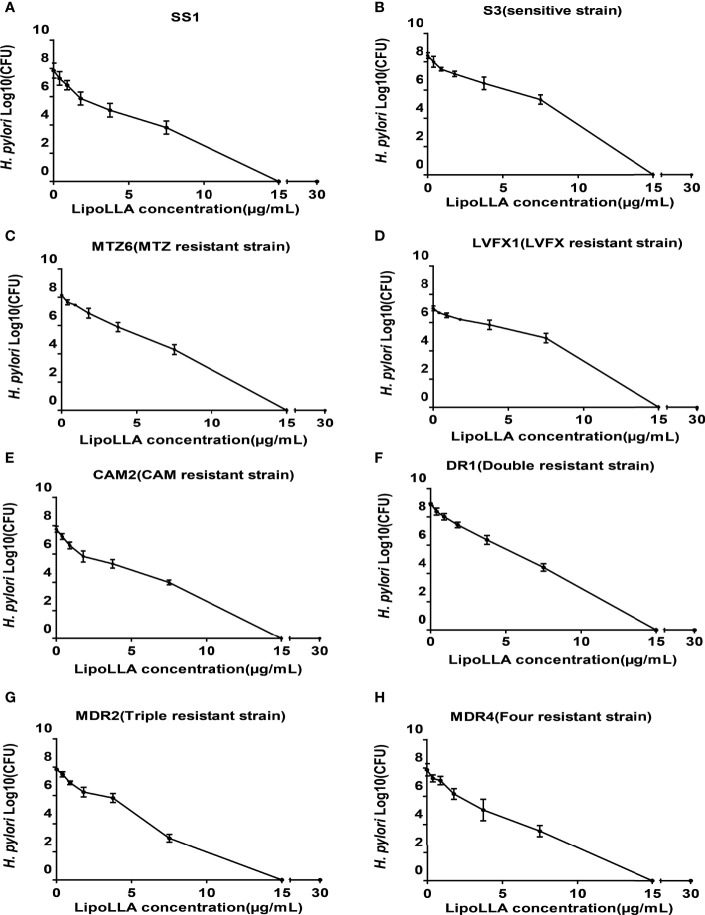
Concentration sterilization curves of LipoLLA against H. pylori strains with different drug resistance. Eight H. pylori strains were co-cultured with LipoLLA respectively to observe the antibacterial activity of LipoLLA against H. pylori with different drug resistance. **(A)** Concentration sterilization curves of LipoLLA against SS1. **(B)** Concentration sterilization curves of LipoLLA against sensitive strain S3. **(C)** Concentration sterilization curves of LipoLLA against MTZ-resistant strain MTZ6. **(D)** Concentration sterilization curves of LipoLLA against LVFX-resistant strain LVFX1. **(E)** Concentration sterilization curves of LipoLLA against CAM-resistant strain CAM2. **(F)** Concentration sterilization curves of LipoLLA against dual drug-resistant strain DR1. **(G)** Concentration sterilization curves of LipoLLA against triple drug-resistant strain MDR2. **(H)** Concentration sterilization curves of LipoLLA against quadruple drug-resistant strain MDR4.

To investigate the bactericidal ability of LipoLLA against *H. pylori*, bactericidal activity curves were prepared for 8 of the tested strains (SS1, sensitive strain S3, MTZ-resistant strain MTZ6, LVFX-resistant strain LVFX1, CAM-resistant strain CAM2, dual drug-resistant strain DR1, triple drug-resistant strain MDR2, and quadruple drug-resistant strain MDR4) (**Figure 2**). After treatment with LipoLLA at different concentrations for 18 h, the number of colonies of the SS1 strain, sensitive strain and drug-resistant strains decreased to some extent. When the concentration of LipoLLA increased to 15μg/mL, no colony growth was observed, suggesting that LipoLLA had a good bactericidal effect on both sensitive and drug-resistant strains of *H. pylori*.

### Influence of pH and Time on the Bactericidal Effect of LipoLLA

**Figure 3:**
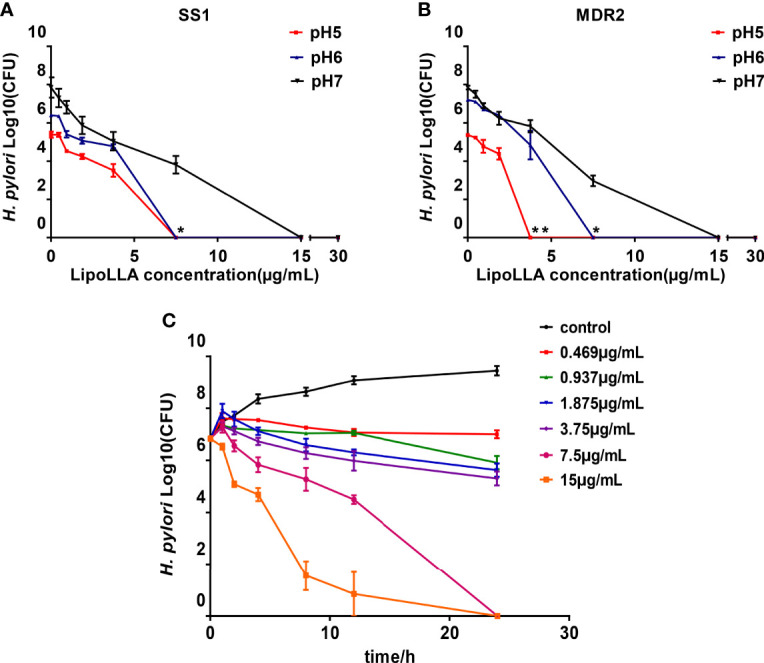
Influence of pH and time on the bactericidal effect of LipoLLA. The concentration bactericidal curve of LipoLLA to SS1 **(A)** and a multidrug resistant strain MDR2 **(B)** at different pH. Time sterilization curves of SS1 with different concentrations of LipoLLA** (C)**. *P < 0.05; **P < 0.01, the number of viable bacterial colonies at pH=5 or pH=6 was significantly different from that at pH=7.

To explore the effect of pH on the bactericidal effect of LipoLLA, we generated a concentration-bactericidal curve for LipoLLA with SS1 and the clinical multidrug-resistant strain MDR2 under different pH values. For SS1, no colony growth was observed when the concentration of LipoLLA reached 15 μg/mL at pH 7; when the concentration of LipoLLA reached 7.5 μg/mL at pH 6 or 5, there was no colony growth, which was significantly different from the result at pH 7 (P<0.05) (**Figure 3A**). For MDR2, no colony growth was observed when the concentration of LipoLLA reached 15 μg/mL at pH 7; when the concentration of LipoLLA reached 3.75 μg/mL at pH 5, no colony growth occurred, and when the concentration reached 7.5 μg/mL at pH 6, no colony growth occurred, which was significantly different from the result at pH 7 (P<0.05) (**Figure 3B**). These results suggested that LipoLLA can completely kill *H. pylori* at a lower concentration at pH 5 or 6 than at pH 7. The time-sterilization curve showed that with the extension of treatment time, the number of viable bacterial colonies decreased gradually. The higher the drug concentration was, the faster the number of viable bacterial colonies decreased (**Figure 3C**). In particular, when the LipoLLA concentration increased to 7.5 μg/mL and 15 μg/mL, the bacterial colonies were completely destroyed after 24 h. These results suggested that the killing effect of LipoLLA on *H. pylori* was time and concentration dependent and that LipoLLA had a better effect on *H. pylori* in a moderately acidic environment.

### Effect of LipoLLA on the Outer Membrane Barrier and Ultrastructure of *H. pylori*

**Figure 4:**
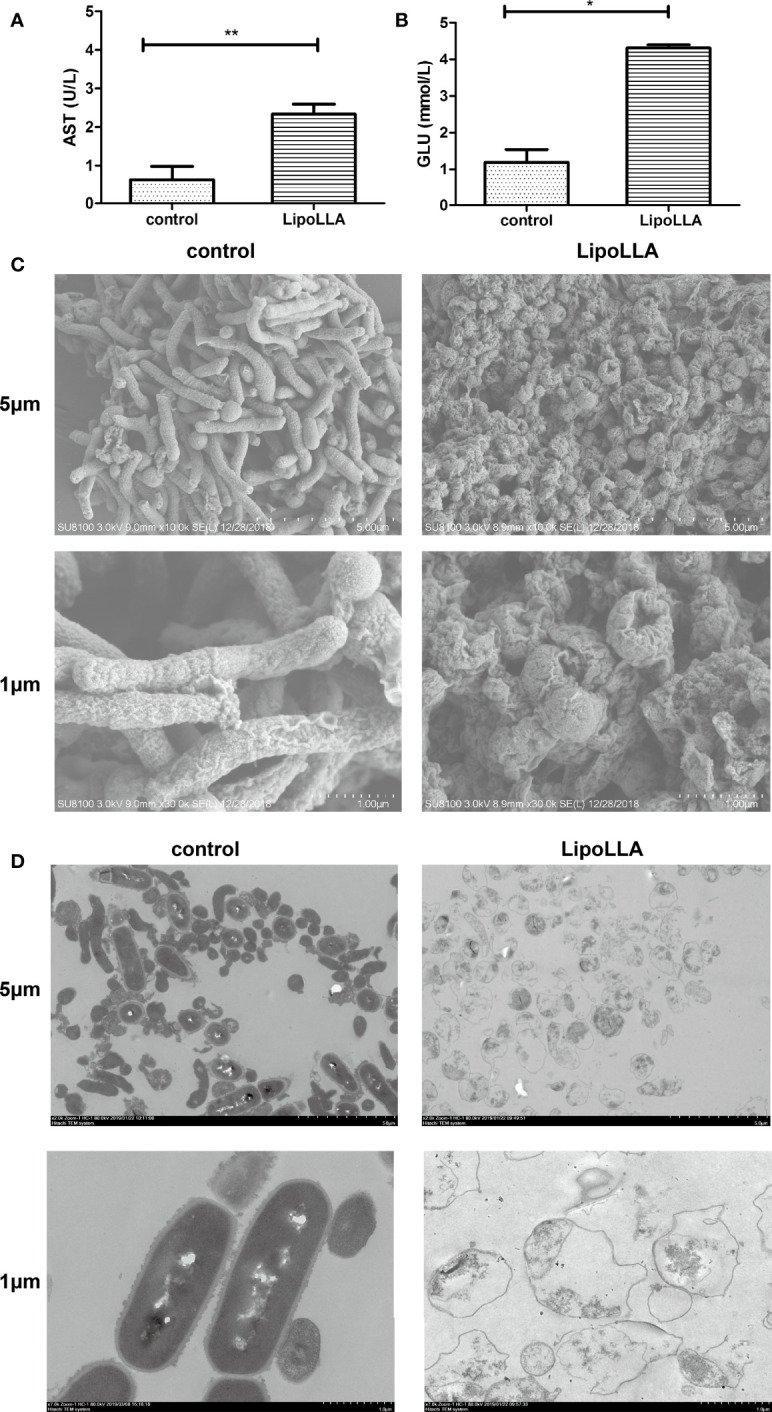
Effect of LipoLLA on the outer membrane barrier and ultrastructure of *H. pylori.* Levels of aspartate aminotransferase **(A)** and glucose **(B)** in SS1 supernatant after LipoLLA treatment, *P < 0.05; **P < 0.01. Scanning electron microscopy images of SS1 after LipoLLA treatment **(C)**. Transmission electron microscopy images of SS1 after LipoLLA treatment **(D)**. Scale bars represent 5 um and 1 um respectively.

The bactericidal mechanism of LipoLLA was explored by content leakage assessment as well as bacterial morphology observation. After treatment with LipoLLA for 24 h, the GLU content in the supernatant of SS1 was significantly increased compared with that in the control group (P<0.05) (**Figure 4A**). The AST content was also significantly higher than that in the control group (P<0.01) (**Figure 4B**). SEM was used to observe the obvious atrophy and adhesion of SS1 bacteria treated with LipoLLA, which was significantly different from the normal morphology (**Figure 4C**). TEM showed that the cell wall of SS1 in the control group was closely connected with the cell membrane, and the cytoplasmic structure was dense, with flagella faintly visible. After treatment with LipoLLA, the outer membrane of SS1 was seriously damaged with an unclear structure, and the cytoplasmic contents were sparse or disappeared (**Figure 4D**). These results indicated that LipoLLA destroyed the outer membrane barrier and ultrastructure of *H. pylori*.

### Bactericidal Effect of LipoLLA on Probiotics

**Figure 5:**
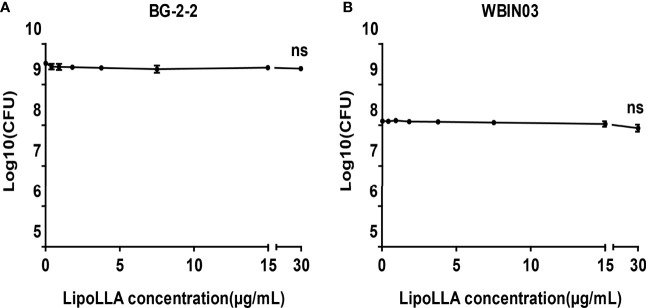
Bactericidal effect of LipoLLA on probiotics. Antibacterial activity of LipoLLA against *Lactobacillus* BG-2-2 **(A)** and *Bifidobacterium* WBIN03 **(B)**. ns, P > 0.05, The viable count after LipoLLA treatment was not significantly different from that in the control group.

*Lactobacillus* BG-2-2 and *Bifidobacterium* WBIN03 were tested to explore the effect of LipoLLA on probiotics. The bactericidal curves showed that the number of viable bacteria of the two strains was on the same order of magnitude as that of the control group when the concentration of LipoLLA increased. When the concentration of LipoLLA increased to 30 μg/mL (at which point *H. pylori* could no longer grow), the number of viable bacteria of *Lactobacillus* BG-2-2 was 2.497 ± 0.266 ×109 CFU/mL, which was not significantly different from that in the control group (3.123 ± 0.464 ×109 CFU/mL) and at other concentrations (P>0.05) (**Figure 5A**). The viable count of *Bifidobacterium* WBIN03 was 8.850 ± 2.486 ×107 CFU/mL at a LipoLLA concentration of 30 μg/mL, which was not significantly different from that in the control group (1.283 ± 0.179 ×108 CFU/mL) and at other concentrations (P>0.05) (**Figure 5B**). These indicated that LipoLLA had no significant effect on *Lactobacillus* and *Bifidobacterium* at the effective anti-*H. pylori* concentration.

### Effect of LipoLLA on the Human Fecal Flora

**Figure 6:**
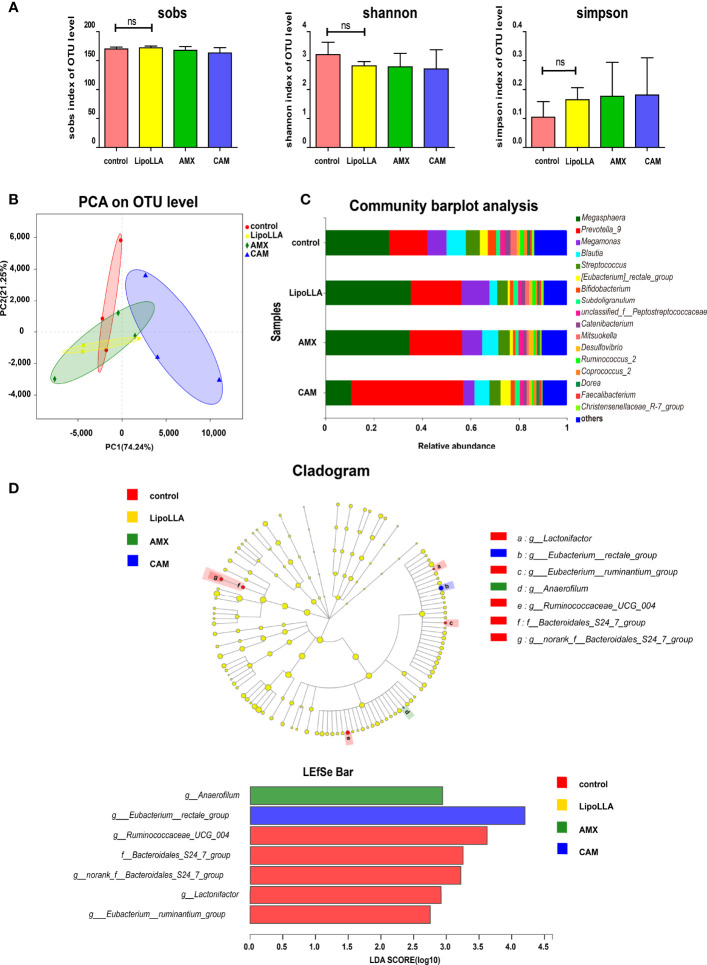
Effect of LipoLLA on the human fecal flora. The alpha diversity indices of the fecal flora after four treatments **(A)**. PCA analysis was predicted to assess Beta diversity of the species after four treatments **(B)**. Analysis of community composition under genus level of four treatment groups **(C)**. The linear discrimination analysis coupled with effect size (LEfSe) analysis of four treatment groups, linear discriminant analysis (LDA analysis) showed the enrichment of different species in each group **(D)**. ns, no significance.

To preliminarily explore whether LipoLLA may affect the human gut microbiota, we collected fecal samples, and the treated samples were analyzed with high-throughput sequencing. The analyzed reads from all 12 fecal samples were clustered into 185 operational taxonomic units (OTUs). The effective sequence length of all samples was distributed between 420 and 460 bp. The rarefaction curve showed that with increasing sample quantity, the total number of species increased, and the number of core species decreased, indicating that the sample was sufficient. The alpha diversity in the fecal flora represented the richness and diversity of species. In this study, we estimated the species richness by the Sobs index, while the Shannon and Simpson indices were used to evaluate the diversity of species. The results showed no significant differences in the three diversity indices between the LipoLLA group and the control (**Figure 6A**). Beta diversity among groups was assessed by principal component analysis (PCA). The results showed that the CAM group remained away from the control, while the LipoLLA group was close to the control group (**Figure 6B**). Together, the results illustrated that LipoLLA had little effect on diversity in the human fecal flora. Next, a bar plot was constructed to study whether the composition of the microbiota was greatly altered. As shown, *Megasphaera* and *Prevotella_*9 were both dominant in all four groups. However, after CAM treatment, the abundance of *Megasphaera* decreased, while that of *Prevotella_*9 increased (**Figure 6C**). The species differences of each group were analyzed by the linear discrimination analysis coupled with effect size (LEfSe) analysis. The results showed that there were significant differences among *Lactonifactor*, *Eubacterium_rectale_group*, *Eubacterium_ruminantium_group*, *Anaerofilum*, *Ruminococcaceae_UCG*_004, *Bacteroidales_S*24_7_*group*, and *norank_f_Bacteroidales_S*24_7_*group*. Linear discriminant analysis (LDA) showed that *Anaerofilum* was significantly enriched in the AMX group (P=0.028). *Eubacterium_rectale_group* was significantly enriched in the CAM group (P=0.033). However, *Lactonifactor* (P=0.029), *Ruminococcaceae_UCG*_004 (P=0.044), *Bacteroidales_S*24_7_group (P=0.033) and *Eubacterium_ruminantium_group* (P=0.040) were significantly enriched in the control group (**Figure 6D**). Together, these findings indicated that LipoLLA caused few significant changes in the human intestinal flora. These results provide the possibility for LipoLLA to be used as a safe anti-*H. pylori* agent.

## Discussion

*H. pylori* infection remains a difficult challenge worldwide, especially in some developing countries (Hooi et al., 2017). Antibiotic regimens have been modified continuously to overcome drug resistance, but the results remain unsatisfactory (Song et al., 2016; Tariq et al., 2020). Obviously, the discovery of an effective *H. pylori* eradication therapy would have global significance. In our study, LipoLLA showed favorable anti-*H. pylori* activity *in vitro* and exhibited little interference with human fecal bacteria.

LipoLLA was prepared by packing LLA into nanoliposomes to overcome the poor hydrophilicity of LLA. As a common drug delivery system, liposomes are deemed to be safe for humans. The liposome formulation can fuse with the cell membrane more stably and deliver LLA to the membrane with less interference with the intracellular pathway than free LLA. Thamphiwatana (Thamphiwatana et al., 2014) found that LipoLLA could reduce colonization by *H. pylori* in mice and ameliorate inflammation, but its antibacterial effect on clinical strains of *H. pylori* was not clear. Our results showed that the MICs of LipoLLA against 30 *H. pylori* clinical isolates were 3.75-15 μg/mL, regardless of whether the strains were sensitive to AMX, MTZ, LVFX and CAM or resistant. LipoLLA showed great antibacterial activity. At present, there is no well-developed monotherapy for *H. pylori* infection. Therefore, according to the MIC of each strain, we determined the combined effect of LipoLLA with AMX, MTZ, LVFX and CAM based on FICI values. The results showed that the MICs of LipoLLA, AMX, MTZ, LVFX and CAM against the 30 clinical isolates of *H. pylori* were all significantly reduced, and the combined effect was synergistic or additive. This provides a basis for the development of a combined antibacterial regimen.

Our study demonstrated that the bactericidal effect of LipoLLA on *H. pylori* was time and concentration dependent and that LipoLLA had a favorable effect on *H. pylori* in a moderately acidic environment. This finding suggested that LipoLLA may be more stable in acidic environments and are suitable for functioning in the stomach. The time dependence of LipoLLA indicates the optimal interval and dosage for clinical application.

Studies have reported that the antibacterial action of free fatty acids targets the cell membrane of bacteria, interfering with the production of bacterial energy by destroying electron transfer chains and oxidative phosphorylation, inhibiting enzyme activity, blocking nutrient absorption, producing peroxide and autooxidation-related degradation products, or directly lysing bacteria (Desbois and Smith, 2010). In our study, we detected the AST content, which is often used as an indicator of changes in bacterial plasma membrane permeability (Zhang et al., 2016), as well as GLU in the supernatant of *H. pylori* after LipoLLA treatment. The increased levels of AST and GLU indicated that the structure of the *H. pylori* plasma membrane and outer membrane was destroyed. In addition, the LipoLLA formulation with a size of ~100 nm had enough surface tension to fuse with the cell membrane, and the phospholipid bilayer structure protected LLA from degradation to enhance the anti-*H. pylori* effect (Thamphiwatana et al., 2014; Jung et al., 2015). The ultrastructural changes in *H. pylori* treated with LipoLLA observed by SEM and TEM showed an atrophied and seriously damaged membrane. It was further confirmed that the special liposome structure made it easy for LipoLLA to fuse with the bacterial membrane and damage the integrity of the membrane, which caused exudation of the bacterial contents and finally resulted in bacterial death. Compared with the action of conventional antibiotics, this anti-*H. pylori* mechanism of LipoLLA was capable of reducing the induction of bacterial drug resistance (Petschow et al., 1996; Thamphiwatana et al., 2014). This feature made LipoLLA strongly destructive against *H. pylori*. LipoLLA produces improved antibacterial effects when used alone, and when used in combination with other antibacterial drugs, the destruction of the outer membrane barrier could also help the other drugs rapidly enter the bacteria, thereby enhancing the antibacterial effect. This may explain why the effects of LipoLLA combined with AMX, MTZ, LVFX, and CAM were synergistic or additive in this study.

Thamphiwatana and colleagues incubated the adenocarcinoma gastric cell line (AGS) with LipoLLA, and reported that there was a negligible release of lactate dehydrogenase, which assessed cell death (Thamphiwatana et al., 2014). And after five consecutive days of administration of LipoLLA in mice, there was no significant change in body weight, stomach tissue section showed no apparent increase in gastric epithelial apoptosis (Thamphiwatana et al., 2014). These results suggested little toxicity of LipoLLA to normal cells when acting against *H. pylori*.

Li found that LipoLLA poses much less effect on the composition and diversity of gastrointestinal microbiota in mice than triple therapy (PPI, AMX and CAM) and therefore has fewer side effects (Li et al., 2018). The impact of the intestinal flora on human health has received increasing attention. Drugs may affect the normal gut flora as well as bacterial resistance (Ianiro et al., 2016; Jackson et al., 2016). Increased levels of drug-resistant bacteria in the intestinal flora could lead to the transfer of resistance genes to pathogenic bacteria and commensal bacteria through horizontal gene transfer, in turn leading to the failure of antimicrobial therapy (Oh et al., 2016). Four years after CAM and MTZ treatment, high level of the macrolide resistance gene erm (B) were found, indicating the antibiotic resistance (Jakobsson et al., 2010). While epidemic spread of erm (B) has been shown to be the cause of increased macrolide resistance in *Streptococcus pyogenes. H. pylori* develops resistance to CAM through point mutation, which clearly affects the efficacy of regimens and determines the first-line treatments of choice (Flores-Treviño et al., 2018). *H. pylori* isolated from patients who later failed the triple therapy showed a greater resistance to CAM. In this study, the concentration-bactericidal curves showed that LipoLLA had no significant effect on the proliferation of *Lactobacillus* BG-2-2 and *Bifidobacterium* WBIN03 when the concentration was 0-30 μg/mL, indicating that LipoLLA effectively killed *H. pylori* without affecting the survival of normal probiotics.

Then, 16S rRNA sequencing demonstrated that LipoLLA has no significant effect on the diversity and species composition of the intestinal flora, indicating that the use of LipoLLA as a potential anti-*H. pylori* drug guarantees for the safety of the intestinal flora to a certain degree. The alpha diversity analysis of 16S rRNA sequencing results showed no change in bacterial species after drug treatments. This is not consistent with previous studies in which AMX or CAM treatment led to a decrease in bacterial diversity (Langdon et al., 2016). The reason may be that the concentration of AMX and CAM that we used was 1 μg/mL, which was constant *in vitro* and did not reach the concentration level that could cause significant changes in bacterial diversity. Another possible reason is that the short duration of drug treatments used may have been insufficient to cause the corresponding changes. Further analysis of the species composition of each treatment group showed that after CAM treatment, *Prevotella* had the highest proportion and became the dominant genus, while the abundance of *Megasphaera* decreased. Studies have shown that a high proportion of *Prevotella* is associated with chronic intestinal inflammation (Ley, 2016) and *Megasphaera* can produce short-chain fatty acids that are beneficial to human health (Cuesta-Zuluaga et al., 2017), indicating damage to the intestinal flora of the CAM group. It was also reported that AMX could decrease the abundance of *Clostridium coccidioides* and *Eubacterium rectale* while increasing the abundance of *Enterobacteriaceae* and the proportion of *Bacteroides* (Barc et al., 2004; Panda et al., 2014). However, there was no significant change in the abundance of these bacteria in this study, possibly because the *in vivo* environment was more complex than the *in vitro* conditions. The internal environment is affected by diet, gastrointestinal diseases, previous drug treatment, individual differences in drug absorption rate and other factors, all of which can have an impact on the therapeutic effect of antibiotics (Oh et al., 2016). The antibacterial activity of fatty acids is relatively broad spectrum. Whereas, in our study, LipoLLA have little effect on intestinal flora of human *in vitro*. This may be related to the selectivity of fatty acids. Different fatty acids may have effects on different bacteria (Desbois and Smith, 2010).

In summary, this study indicated that LipoLLA had an antibacterial effect on *H. pylori* strains *in vitro*, exhibiting a synergistic or additive effect when combined with common anti-*H. pylori* drugs. The mechanism of action of LipoLLA against *H. pylori* involved destruction of the outer membrane barrier of bacteria, which made it difficult for bacteria to develop drug resistance. In addition, this study found that LipoLLA had no significant effect on the intestinal flora *in vitro*, providing preliminarily a basic guarantee for the safety of LipoLLA.

## Data Availability Statement

The original contributions presented in the study are publicly available. This data can be found here: https://www.ncbi.nlm.nih.gov/sra/PRJNA814527, accession number: PRJNA814527.

## Ethics Statement

The research protocol was approved by the Ethics Committee of the First Affiliated Hospital of Nanchang University (IRB 2018-116 and IRB 2014-032). The participant provided written informed consent to participate in this study. Written informed consent was obtained from the individual(s) for the publication of any potentially identifiable images or data included in this article.

## Author Contributions

YX and YF designed the study. YW and SW acquired and analyzed the data, drafted the manuscript. LW, YW, and DL revised contents critically. All authors contributed to the article and approved the submitted version.

## Funding

Funding was provided by grants from the National Natural Science Foundation of China (No. 81970502, No. 81860107, No.82060109), the Science and Technology Projects of Jiangxi Province (20203BBG73051), Science and technology research project of education department of Jiangxi province in 2018(GJJ180047), and the supervision project of Jiangxi Provincial Drug Administration (2020JS16).

## Conflict of Interest

The authors declare that the research was conducted in the absence of any commercial or financial relationships that could be construed as a potential conflict of interest.

## Publisher’s Note

All claims expressed in this article are solely those of the authors and do not necessarily represent those of their affiliated organizations, or those of the publisher, the editors and the reviewers. Any product that may be evaluated in this article, or claim that may be made by its manufacturer, is not guaranteed or endorsed by the publisher.
