# Supplementary material for: The Activity of Liposomal Linolenic Acid Against Helicobacter pylori In Vitro and Its Impact on Human Fecal Bacteria
Source: Front Cell Infect Microbiol. 2022 May 17;12:865320. doi: 10.3389/fcimb.2022.865320 (PMC9152453; doi:10.3389/fcimb.2022.865320)
Supplement: Supplementary file 1 [file DataSheet_1.docx]

Supplementary Material

Table S1 The MIC of four antibacterial drugs amoxicillin, metronidazole, levofloxacin and clarithromycin on the quality control strain ATCC43504

|  | MIC(μg/mL) | | | |
| --- | --- | --- | --- | --- |
|  | AMX | MTZ | LVFX | CAM |
| ATCC43504 | 0.032 | 128 | 0.064 | 0.125 |
| the range of quality control | 0.016-0.12 | 64-256 | 0.016-0.25 | 0.016-0.12 |

AMX: amoxicillin; MTZ: metronidazole; LVFX: levofloxacin; CAM: clarithromycin.

**Table S2 The combined effect of LipoLLA with amoxicillin**

| Strain | MIC(μg/mL) alone | |  | MIC(μg/mL) combination | | FICI | outcome |
| --- | --- | --- | --- | --- | --- | --- | --- |
|  | AMX | LipoLLA |  | AMX | LipoLLA |  |  |
| S1 | 0.016 | 7.500 |  | 0.001 | 0.234 | 0.094 | Synergistic |
| S2 | 0.016 | 7.500 |  | 0.001 | 0.234 | 0.094 | Synergistic |
| S3 | 0.016 | 7.500 |  | 0.002 | 0.234 | 0.156 | Synergistic |
| S4 | 0.016 | 7.500 |  | 0.002 | 0.234 | 0.156 | Synergistic |
| MTZ1 | 0.016 | 7.500 |  | 0.002 | 0.234 | 0.156 | Synergistic |
| MTZ4 | 0.016 | 7.500 |  | 0.001 | 0.234 | 0.094 | Synergistic |
| MTZ5 | 0.016 | 7.500 |  | 0.002 | 0.234 | 0.156 | Synergistic |
| MTZ6 | 0.016 | 7.500 |  | 0.002 | 0.234 | 0.156 | Synergistic |
| LVFX2 | 0.016 | 7.500 |  | 0.002 | 0.234 | 0.156 | Synergistic |
| LVFX3 | 0.016 | 15.000 |  | 0.002 | 0.469 | 0.156 | Synergistic |
| LVFX4 | 0.016 | 15.000 |  | 0.002 | 0.937 | 0.187 | Synergistic |
| LVFX5 | 0.016 | 7.500 |  | 0.002 | 0.937 | 0.250 | Synergistic |
| CAM3 | 0.016 | 7.500 |  | 0.002 | 0.234 | 0.156 | Synergistic |
| CAM4 | 0.016 | 7.500 |  | 0.002 | 0.234 | 0.156 | Synergistic |
| CAM5 | 0.016 | 7.500 |  | 0.002 | 0.234 | 0.156 | Synergistic |
| DR1 | 0.016 | 7.500 |  | 0.002 | 0.234 | 0.156 | Synergistic |
| DR2 | 0.016 | 3.750 |  | 0.002 | 0.234 | 0.187 | Synergistic |
| DR3 | 0.016 | 7.500 |  | 0.002 | 0.234 | 0.156 | Synergistic |
| MDR1 | 0.016 | 3.750 |  | 0.001 | 0.117 | 0.094 | Synergistic |
| MDR2 | 0.016 | 7.500 |  | 0.001 | 0.937 | 0.187 | Synergistic |
| MDR3 | 0.016 | 7.500 |  | 0.001 | 0.234 | 0.094 | Synergistic |
| S5 | 0.032 | 7.500 |  | 0.001 | 0.469 | 0.094 | Synergistic |
| MTZ3 | 0.032 | 7.500 |  | 0.001 | 0.469 | 0.094 | Synergistic |
| LVFX1 | 0.032 | 15.000 |  | 0.002 | 0.234 | 0.078 | Synergistic |
| CAM1 | 0.032 | 7.500 |  | 0.001 | 0.234 | 0.062 | Synergistic |
| CAM2 | 0.032 | 7.500 |  | 0.001 | 0.234 | 0.062 | Synergistic |
| MTZ2 | 0.063 | 7.500 |  | 0.002 | 0.234 | 0.063 | Synergistic |
| MDR4 | 0.063 | 7.500 |  | 0.004 | 0.234 | 0.095 | Synergistic |
| MDR5 | 0.125 | 7.500 |  | 0.004 | 0.234 | 0.063 | Synergistic |
| MDR6 | 1.000 | 7.500 |  | 0.063 | 0.234 | 0.094 | Synergistic |
|  |  |  |  | *** | *** |  |  |
| AMX, amoxicillin; ****P*<0.001, The MICs of LipoLLA and AMX against *H. pylori* were significantly decreased after combination. | | | | | | | |

**Table S3 The combined effect of LipoLLA with metronidazole**

| strain | MIC(μg/mL) alone | |  | MIC(μg/mL) combination | | FICI | outcome |  |
| --- | --- | --- | --- | --- | --- | --- | --- | --- |
|  | MTZ | LipoLLA |  | MTZ | LipoLLA |  |  |  |
| LVFX1 | 0.500 | 15.000 |  | 0.016 | 0.117 | 0.040 | Synergistic |  |
| S1 | 1.000 | 7.500 |  | 0.032 | 0.117 | 0.048 | Synergistic |  |
| S2 | 1.000 | 7.500 |  | 0.032 | 0.117 | 0.048 | Synergistic |  |
| S4 | 1.000 | 7.500 |  | 0.032 | 0.117 | 0.048 | Synergistic |  |
| LVFX5 | 1.000 | 7.500 |  | 0.063 | 3.750 | 0.563 | Additive |  |
| LVFX3 | 2.000 | 15.000 |  | 0.063 | 0.117 | 0.039 | Synergistic |  |
| CAM3 | 2.000 | 7.500 |  | 0.063 | 0.117 | 0.047 | Synergistic |  |
| CAM4 | 2.000 | 7.500 |  | 0.063 | 0.117 | 0.047 | Synergistic |  |
| CAM5 | 2.000 | 7.500 |  | 0.250 | 0.117 | 0.141 | Synergistic |  |
| DR1 | 2.000 | 7.500 |  | 0.063 | 0.117 | 0.047 | Synergistic |  |
| S5 | 2.000 | 7.500 |  | 0.063 | 0.117 | 0.047 | Synergistic |  |
| CAM1 | 2.000 | 7.500 |  | 0.063 | 0.117 | 0.047 | Synergistic |  |
| CAM2 | 2.000 | 7.500 |  | 0.063 | 0.117 | 0.047 | Synergistic |  |
| S3 | 4.000 | 7.500 |  | 0.125 | 0.937 | 0.156 | Synergistic |  |
| LVFX2 | 4.000 | 7.500 |  | 0.125 | 0.937 | 0.156 | Synergistic |  |
| LVFX4 | 4.000 | 15.000 |  | 0.125 | 0.234 | 0.047 | Synergistic |  |
| MTZ1 | 8.000 | 7.500 |  | 0.250 | 0.937 | 0.156 | Synergistic |  |
| DR2 | 8.000 | 3.750 |  | 0.250 | 0.117 | 0.062 | Synergistic |  |
| MDR1 | 8.000 | 3.750 |  | 0.250 | 0.117 | 0.062 | Synergistic |  |
| MTZ2 | 16.000 | 7.500 |  | 1.000 | 0.234 | 0.094 | Synergistic |  |
| MTZ4 | 32.000 | 7.500 |  | 2.000 | 0.234 | 0.094 | Synergistic |  |
| DR3 | 32.000 | 7.500 |  | 1.000 | 0.234 | 0.062 | Synergistic |  |
| MDR2 | 32.000 | 7.500 |  | 2.000 | 0.234 | 0.094 | Synergistic |  |
| MDR3 | 32.000 | 7.500 |  | 1.000 | 0.117 | 0.047 | Synergistic |  |
| MTZ3 | 32.000 | 7.500 |  | 1.000 | 0.117 | 0.047 | Synergistic |  |
| MDR4 | 32.000 | 7.500 |  | 1.000 | 0.117 | 0.047 | Synergistic |  |
| MTZ5 | 128.000 | 7.500 |  | 4.000 | 0.117 | 0.047 | Synergistic |  |
| MTZ6 | 256.000 | 7.500 |  | 8.000 | 0.234 | 0.062 | Synergistic |  |
| MDR5 | 256.000 | 7.500 |  | 4.000 | 0.117 | 0.031 | Synergistic |  |
| MDR6 | 256.000 | 7.500 |  | 8.000 | 0.117 | 0.047 | Synergistic |  |
|  |  |  |  | *** | *** |  |  |  |
| MTZ, metronidazole; ****P*<0.001, The MICs of LipoLLA and MTZ against *H. pylori* were significantly decreased after combination. | | | | | | | |  |

**Table S4 The combined effect of LipoLLA with levofloxacin**

| strain | MIC(μg/mL) alone | |  | MIC(μg/mL) combination | | FICI | outcome |
| --- | --- | --- | --- | --- | --- | --- | --- |
|  | LVFX | LipoLLA |  | LVFX | LipoLLA |  |  |
| S4 | 0.032 | 7.5 |  | 0.001 | 0.234 | 0.062 | Synergistic |
| S3 | 0.032 | 7.5 |  | 0.001 | 1.800 | 0.271 | Synergistic |
| MTZ1 | 0.032 | 7.5 |  | 0.001 | 0.234 | 0.062 | Synergistic |
| CAM4 | 0.063 | 7.5 |  | 0.004 | 0.234 | 0.095 | Synergistic |
| S2 | 0.125 | 7.5 |  | 0.004 | 0.234 | 0.063 | Synergistic |
| CAM3 | 0.125 | 7.5 |  | 0.004 | 0.234 | 0.063 | Synergistic |
| CAM5 | 0.125 | 7.5 |  | 0.004 | 0.234 | 0.063 | Synergistic |
| CAM1 | 0.125 | 7.5 |  | 0.004 | 0.234 | 0.063 | Synergistic |
| MTZ2 | 0.125 | 7.5 |  | 0.004 | 0.234 | 0.063 | Synergistic |
| S1 | 0.25 | 7.5 |  | 0.008 | 0.234 | 0.063 | Synergistic |
| S5 | 0.25 | 7.5 |  | 0.008 | 1.800 | 0.272 | Synergistic |
| CAM2 | 0.25 | 7.5 |  | 0.008 | 0.234 | 0.063 | Synergistic |
| DR2 | 0.25 | 3.75 |  | 0.032 | 0.469 | 0.253 | Synergistic |
| MTZ4 | 0.25 | 7.5 |  | 0.008 | 1.800 | 0.272 | Synergistic |
| MTZ3 | 0.25 | 7.5 |  | 0.008 | 0.234 | 0.063 | Synergistic |
| MTZ5 | 0.25 | 7.5 |  | 0.008 | 0.234 | 0.063 | Synergistic |
| MTZ6 | 0.25 | 7.5 |  | 0.008 | 0.234 | 0.063 | Synergistic |
| DR3 | 1 | 7.5 |  | 0.063 | 0.234 | 0.094 | Synergistic |
| DR1 | 2 | 7.5 |  | 0.063 | 0.234 | 0.063 | Synergistic |
| MDR4 | 2 | 7.5 |  | 0.063 | 0.234 | 0.063 | Synergistic |
| LVFX1 | 32 | 15 |  | 4.000 | 0.234 | 0.141 | Synergistic |
| MDR3 | 32 | 7.5 |  | 4.000 | 0.234 | 0.156 | Synergistic |
| LVFX5 | 128 | 7.5 |  | 4.000 | 0.469 | 0.094 | Synergistic |
| LVFX3 | 128 | 15 |  | 4.000 | 0.469 | 0.063 | Synergistic |
| LVFX2 | 128 | 7.5 |  | 4.000 | 0.469 | 0.094 | Synergistic |
| LVFX4 | 128 | 15 |  | 4.000 | 0.469 | 0.063 | Synergistic |
| MDR1 | 128 | 3.75 |  | 4.000 | 0.234 | 0.094 | Synergistic |
| MDR2 | 128 | 7.5 |  | 4.000 | 0.234 | 0.062 | Synergistic |
| MDR5 | 128 | 7.5 |  | 4.000 | 0.234 | 0.062 | Synergistic |
| MDR6 | 128 | 7.5 |  | 4.000 | 0.234 | 0.062 | Synergistic |
|  |  |  |  | *** | *** |  |  |
| LVFX, levofloxacin; ****P*<0.001, The MICs of LipoLLA and LVFX against *H. pylori* were significantly decreased after combination. | | | | | | | |

**Table S5 The combined effect of LipoLLA with clarithromycin**

| strain | MIC(μg/mL) alone | |  | MIC(μg/mL) combination | | FICI | Outcome |
| --- | --- | --- | --- | --- | --- | --- | --- |
|  | CAM | LipoLLA |  | CAM | LipoLLA |  |  |
| LVFX4 | 0.016 | 15 |  | 0.002 | 0.117 | 0.133 | Synergistic |
| S4 | 0.063 | 7.5 |  | 0.002 | 0.234 | 0.063 | Synergistic |
| MTZ1 | 0.063 | 7.5 |  | 0.002 | 3.750 | 0.532 | Additive |
| S2 | 0.063 | 7.5 |  | 0.002 | 0.234 | 0.063 | Synergistic |
| MTZ2 | 0.063 | 7.5 |  | 0.002 | 0.234 | 0.063 | Synergistic |
| S1 | 0.063 | 7.5 |  | 0.002 | 0.234 | 0.063 | Synergistic |
| S5 | 0.063 | 7.5 |  | 0.002 | 0.234 | 0.063 | Synergistic |
| MTZ4 | 0.063 | 7.5 |  | 0.002 | 0.234 | 0.063 | Synergistic |
| MTZ5 | 0.063 | 7.5 |  | 0.002 | 0.234 | 0.063 | Synergistic |
| MTZ6 | 0.063 | 7.5 |  | 0.002 | 0.234 | 0.063 | Synergistic |
| DR3 | 0.063 | 7.5 |  | 0.002 | 0.234 | 0.063 | Synergistic |
| LVFX5 | 0.063 | 7.5 |  | 0.002 | 0.234 | 0.063 | Synergistic |
| LVFX3 | 0.063 | 15 |  | 0.004 | 0.234 | 0.079 | Synergistic |
| LVFX2 | 0.063 | 7.5 |  | 0.002 | 0.234 | 0.063 | Synergistic |
| S3 | 0.125 | 7.5 |  | 0.004 | 0.234 | 0.063 | Synergistic |
| LVFX1 | 0.125 | 15 |  | 0.004 | 0.234 | 0.048 | Synergistic |
| MTZ3 | 0.5 | 7.5 |  | 0.032 | 0.117 | 0.080 | Synergistic |
| DR2 | 1 | 3.75 |  | 0.032 | 1.800 | 0.512 | Additive |
| DR1 | 1 | 7.5 |  | 0.032 | 1.875 | 0.282 | Synergistic |
| CAM1 | 8 | 7.5 |  | 0.250 | 0.117 | 0.047 | Synergistic |
| CAM2 | 8 | 7.5 |  | 0.250 | 0.117 | 0.047 | Synergistic |
| MDR3 | 8 | 7.5 |  | 0.250 | 0.234 | 0.062 | Synergistic |
| MDR1 | 8 | 3.75 |  | 0.250 | 0.117 | 0.062 | Synergistic |
| MDR2 | 8 | 7.5 |  | 0.250 | 0.234 | 0.062 | Synergistic |
| MDR5 | 8 | 7.5 |  | 0.250 | 3.750 | 0.531 | Additive |
| MDR4 | 16 | 7.5 |  | 0.500 | 3.750 | 0.531 | Additive |
| CAM4 | 256 | 7.5 |  | 8.000 | 0.117 | 0.047 | Synergistic |
| CAM3 | 256 | 7.5 |  | 8.000 | 0.117 | 0.047 | Synergistic |
| CAM5 | 256 | 7.5 |  | 8.000 | 0.117 | 0.047 | Synergistic |
| MDR6 | 256 | 7.5 |  | 8.000 | 0.117 | 0.047 | Synergistic |
|  |  |  |  |  | *** | *** |  |

CAM, clarithromycin; ****P*<0.001, The MICs of LipoLLA and CAM against *H. pylori* were significantly decreased after combination.

**
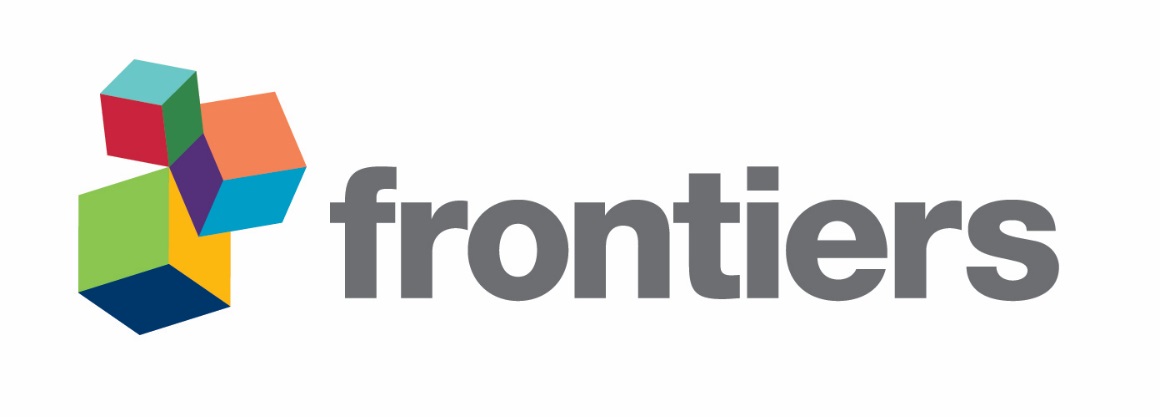
**
